# Therapeutic Notes

**Published:** 1896-09-26

**Authors:** 


### THERAPEUTIC NOTES.

### FORMALIN GELATINE, A NEW ANTISEPTIC POWDER.

On August 1st we gave an account of the various modern applications of formalin, and on July 25th and August 15th a method of disinfecting rooms by the vapour of formaldehyde; but so far no complete account has appeared in this journal of the preparation of the new antiseptic application introduced by Schleich,[1] and prepared by the action of formaldehyde vapour on gelatine. The method of preparation is as follows: A strong solution of gelatine is allowed to dry over formalin vapour, and the desiccated body so formed no longer has the ordinary properties of gelatine; it is a permanent substance, resistant, hard, and transparent; cannot be melted by dry or moist heat, by organic or inorganic acids, or by alkalies. The combined formalin offers no antiseptic properties to bodies which come in contact with it. The gelatine can, however, become decomposed, and the formalin set free by the process of cell activity, or by the fermentative action of pepsin and hydrochloric acid.

If, therefore, formalin gelatine be applied in powdered form to the healthy surface of a wound, the physiological cell activity breaks down the combination of gelatine and the antiseptic, and a continuous stream of formalin vapour is liberated, and the wound preserved in a absolutely aseptic condition. If in contact with dead tissues—necrosed bone, for instance—this action does not proceed, but by the addition of pepsin and hydrochloric acid, Schleich has been able to ensure the desired liberation of the antiseptic.

For plastic operations this new preparation seems eminently suited, for it has been found that if a ball of the material be inserted into the peritoneal cavity of a rabbit, and let *in situ* for some length of time, it becomes completely organised and converted into fibrous connective tissue; for bone grafting it seems equally well adapted; a mould of the shaft of a bone may be inserted in the periosteum, and there left until it becomes organised. The addition of calcium salts appears to hasten the procedure.

The continuous antiseptic action of formalin gelatine is analogous to that of salol, which, in the alimentary tract, or in contact with living tissue, becomes decomposed into phenol and salicylate of soda; and to that of salicylate of soda when exposed to the high carbonic acid tension of inflamed tissues; or, again, it appears similar to the continued antiseptic action of iodoform, which, under like conditions, owes its antiseptic qualities to the liberation of free iodine.

[1] Therapeut. Monatshelf. February, 1896.

